# Supplementary material for: Resolving the full spectrum of human genome variation using Linked-Reads
Source: Genome Res. 2019 Apr;29(4):635–45. doi: 10.1101/gr.234443.118 (PMC6442396; doi:10.1101/gr.234443.118)
Supplement: Supplemental Material [file supp_gr.234443.118_Supplemental_longranger-2.2.zip › longranger-2.2.tar.bz2!/lr2.2-public/lib/stan/cmdstan/stan/doc/api/html/functions_vars_l.html]

Stan: Class Members - Variables


|  |  |
| --- | --- |
|  | Stan  2.5.0  probability, sampling & optimization |


- Main Page
- Related Pages
- Namespaces
- Classes
- Files

- Class List
- Class Index
- Class Hierarchy
- Class Members

- All
- Functions
- Variables
- Typedefs
- Enumerator
- Related Functions

- \_
- a
- b
- c
- d
- e
- f
- g
- h
- i
- k
- l
- m
- n
- o
- p
- q
- r
- s
- t
- u
- v
- w
- x
- y
- z


All Classes Namespaces Files Functions Variables Typedefs Enumerations Enumerator Friends Macros Pages

### - l -

- L\_
  : stan::mcmc::base\_static\_hmc< M, P, H, I, BaseRNG >
- left
  : stan::gm::binary\_op
- local\_decl\_
  : stan::gm::statements
- local\_var\_decls\_r
  : stan::gm::statement\_grammar< Iterator >
- log\_prob\_
  : stan::gm::increment\_log\_prob\_statement
- log\_u
  : stan::mcmc::nuts\_util
- low\_
  : stan::gm::range


---

[ Stan Home Page ]

*© 2011–2014,
Stan Development Team.*
